# Advancing the argument for validity of the Alberta Context Tool with healthcare aides in residential long-term care

**DOI:** 10.1186/1471-2288-11-107

**Published:** 2011-07-18

**Authors:** Carole A Estabrooks, Janet E Squires, Leslie A Hayduk, Greta G Cummings, Peter G Norton

**Affiliations:** 1 Faculty of Nursing, University of Alberta, Edmonton, Canada; 2 Clinical Epidemiology Program, Ottawa Hospital Research Institute, Ottawa, Canada; 3 Department of Sociology, University of Alberta, Edmonton, Canada; 4 Department of Family Medicine, University of Calgary, Calgary, Canada

## Abstract

**Background:**

Organizational context has the potential to influence the use of new knowledge. However, despite advances in understanding the theoretical base of organizational context, its measurement has not been adequately addressed, limiting our ability to quantify and assess context in healthcare settings and thus, advance development of contextual interventions to improve patient care. We developed the Alberta Context Tool (the ACT) to address this concern. It consists of 58 items representing 10 modifiable contextual concepts. We reported the initial validation of the ACT in 2009. This paper presents the second stage of the psychometric validation of the ACT.

**Methods:**

We used the *Standards for Educational and Psychological Testing *to frame our validity assessment. Data from 645 English speaking healthcare aides from 25 urban residential long-term care facilities (nursing homes) in the three Canadian Prairie Provinces were used for this stage of validation. In this stage we focused on: (1) advanced aspects of internal structure (e.g., confirmatory factor analysis) and (2) relations with other variables validity evidence. To assess reliability and validity of scores obtained using the ACT we conducted: Cronbach's alpha, confirmatory factor analysis, analysis of variance, and tests of association. We also assessed the performance of the ACT when individual responses were aggregated to the care unit level, because the instrument was developed to obtain unit-level scores of context.

**Results:**

Item-total correlations exceeded acceptable standards (> 0.3) for the majority of items (51 of 58). We ran three confirmatory factor models. Model 1 (all ACT items) displayed unacceptable fit overall and for five specific items (1 item on *adequate space for resident care *in the Organizational Slack-Space ACT concept and 4 items on use of electronic resources in the Structural and Electronic Resources ACT concept). This prompted specification of two additional models. Model 2 used the 7 scaled ACT concepts while Model 3 used the 3 count-based ACT concepts. Both models displayed substantially improved fit in comparison to Model 1. Cronbach's alpha for the 10 ACT concepts ranged from 0.37 to 0.92 with 2 concepts performing below the commonly accepted standard of 0.70. Bivariate associations between the ACT concepts and instrumental research utilization levels (which the ACT should predict) were statistically significant at the 5% level for 8 of the 10 ACT concepts. The majority (8/10) of the ACT concepts also showed a statistically significant trend of increasing mean scores when arrayed across the lowest to the highest levels of instrumental research use.

**Conclusions:**

The validation process in this study demonstrated additional empirical support for construct validity of the ACT, when completed by healthcare aides in nursing homes. The overall pattern of the data was consistent with the structure hypothesized in the development of the ACT and supports the ACT as an appropriate measure for assessing organizational context in nursing homes. Caution should be applied in using the one space and four electronic resource items that displayed misfit in this study with healthcare aides until further assessments are made.

## Background

Organizational context refers to "...the environment or setting in which people receive healthcare services, or in the context of getting research evidence into practice, the environment or setting in which the proposed change is to be implemented" [1] (page 299). Health services researchers are increasingly aware of the central role that organizational context plays in knowledge translation (the uptake of research evidence) by healthcare providers, and the potential role of context in improving patient, staff, and system outcomes. As a result, a growing body of knowledge on organizational context that crosses multiple disciplines and sectors is emerging [2-9]. Despite the advances in understanding the theoretical base of organizational context, its measurement has not been adequately addressed. This limits our ability to quantify and assess context in healthcare settings and thereby hinders the development and assessment of context-based interventions designed to improve patient care, and staff and system outcomes. The Alberta Context Tool (the ACT) was developed in 2006 to address this concern.

The ACT measures organizational context in complex healthcare settings by assessing care providers' and/or care managers' perceptions of context related to a specific patient/resident care unit or organization (e.g., hospital or nursing home) [10]. The instrument is premised on knowledge translation theory, specifically: (1) the *Promoting Action on Research Implementation in Health Services (PARiHS) *framework of research implementation, which asserts that successful implementation of research evidence is a function of interplay between three factors: context, facilitation and evidence [11,12] and (2) related literature in the fields of organizational science, research implementation, and knowledge translation [4,5,13]. Principles that informed the development of the ACT included brevity (it could be completed in 10 minutes or less) and a focus on potentially modifiable elements of context. Further details on the development of the ACT are published elsewhere [10].

The instrument currently exists in four versions (acute care adult hospitals, acute care pediatric hospitals, long-term care [nursing homes], and home care) and six forms (healthcare aides, nurses, physicians, allied health providers, practice specialists, and care managers). The versions and forms are substantively the same and differ only in the structure of item stems and examples of concepts. Depending on the form, the ACT contains 56-58 items which reflect 10 concepts of organizational context: (1) leadership, (2) culture, (3) evaluation (feedback processes), (4) social capital, (5) informal interactions, (6) formal interactions, (7) structural and electronic resources, (8) organizational slack-staff, (9) organizational slack-space, and (10) organizational slack-time. The long-term care healthcare aide version assessed in this paper contains 58 items. Definitions of the ACT concepts, along with our hypotheses about their association to the uptake of research evidence, are presented in Table 1.

**Table 1:** Concepts in the ACT Survey

Concept	Definition	Hypothesis	Sample item
Leadership ^1^	The actions of formal leaders in an organization (unit) to influence change and excellence in practice, items generally reflect emotionally intelligent leadership	**H1**: Care providers who perceive more positive (emotionally intelligent) unit leadership report higher research use	The leader calmly handles stressful situations

Culture ^1^	The way that "we do things' in our organizations and work units; items generally reflect a supportive work culture	**H2: **Care providers who perceive a more positive unit culture report higher research use	My organization effectively balances best practice and productivity

Evaluation ^1^	The process of using data to assess group/team performance and to achieve outcomes in organizations or units (i.e., evaluation)	**H3: **Care providers who perceive a larger number of unit feedback mechanisms report higher research use	Our team routinely monitors our performance with respect to the action plans

Social Capital ^1^	The stock of active connections among people. These connections are of three types: bonding, bridging, and linking	**H4: **Care providers who perceive more positive unit social capital activities report higher research use	People in the group share information with others in the group

Informal Interactions ^2^	Informal exchanges that occur between individuals working within an organization (unit) that can promote the transfer of knowledge	**H5: **Care providers who perceive a larger number of informal unit interactions report higher research use	How often do you interact with people in the following roles or positions? - Someone who *champions *research and its use in practice

Formal Interactions ^2^	Formal exchanges that occur between individuals working within an organization (unit) through scheduled activities that can promote the transfer of knowledge	**H6: **Care providers who perceive a larger number of formal unit interactions report higher research use	How often do these activities occur? -Team meetings

Structural/Electronic Resources ^3^	The structural and electronic elements of an organization (unit) that facilitate the ability to assess and use knowledge	**H7: **Care providers who perceive a larger number of unit structural and electronic resources report higher research use	How often do you use/attend the following? - Notice Boards

Organizational Slack	The cushion of actual or potential resources which allows an organization (unit) to adapt successfully to internal pressures for adjustments or to external pressures for changes		
Staff ^1^		**H8: **Care providers who perceive sufficient unit staffing levels report higher research use	Enough staff to deliver quality care
Space ^1^		**H9: **Care providers who perceive having sufficient space on their unit report higher research use	Use of designated space
Time ^1^		**H10: **Care providers who perceive having sufficient time on their unit report higher research use	Time to do something extra for patients

^1 ^= Scale: 1-strongly disagree; 2-disagree; 3-neither agree nor disagree; 4-agree; 5-strongly agree

^2 ^= Scale: 1-never; 2-rarely; 3-ocasionally; 4-frequently; 5-almost always

^3 ^= Scale: 1-never; 2-rarely; 3-ocasionally; 4-frequently; 5-almost always; 6- not available.

Initial validation of the ACT was conducted on scores obtained using the 56-item instrument in a national, multi-site study of pediatric nurse professionals (N = 752 responses). In that study, a principal components analysis indicated a 13-factor solution (accounting for 59.26% of the variance and covariance in 'organizational context') [10]. Initial construct validity was further supported with statistically significant correlations between the ACT factors and instrumental research utilization (i.e., the concrete application of research findings in practice, for example, use of guidelines). Adequate internal consistency reliability with Cronbach's alpha coefficients ranging from 0.54 to a 0.91 for the 13 factors was also reported. The **purpose **of the present study is to advance a validity argument for the ACT by assessing the reliability, acceptability, and validity of scores obtained with the instrument when completed by a somewhat different population, namely healthcare aides in residential long-term care settings (nursing homes).

## Methods

### Design

The data analyzed in this paper are from the Translating Research in Elder Care (TREC) study [14]. TREC is a multi-level longitudinal descriptive study aimed at identifying modifiable characteristics of organizational context in nursing homes that are associated with the uptake of research evidence by care providers and care managers, and the subsequent impact of this uptake on resident health (e.g., number of falls) and staff outcomes (e.g., burnout). TREC is situated in 36 nursing homes in the three Canadian Prairie Provinces of Alberta, Saskatchewan, and Manitoba, and is comprised of two interrelated projects and a series of pilot studies [14]. The two major projects are: (1) TREC Project One - Building context, an organizational monitoring system in long-term care [15], and (2) TREC Project Two-Building context, a case study program in long-term care [16]. Analyses in this paper utilize data from TREC Project One.

### Sampling

We drew two nursing home samples. The first sample consisted of 30 urban nursing homes, and the second of six rural nursing homes. We selected the 30 urban nursing homes using stratified random sampling. All urban nursing homes meeting the TREC inclusion criteria (see Additional File 1) were stratified according to three factors: (1) healthcare region (within province), (2) operational model (public, private, voluntary), and (3) size (small: 35 to 149 beds, large: ≥150 beds), producing six lists of eligible nursing homes per region. We then used stratified random sampling to select the 30 nursing homes. The analyses presented here use data from 25 of the 30 urban nursing homes. We excluded the six rural nursing homes in the sample (which were a convenience sample) because of urban-rural differences in context (as assessed by the ACT) and smaller facility size. In addition, the rural nursing homes tended to have only one unit. We also excluded five urban nursing homes that had only one unit, as more than one unit is required to run the aggregation statistics reported here. The team used a volunteer, census-like sampling technique to recruit individual participants within the nursing homes.

### Data Collection

We collected data in TREC Project One at three levels: (1) facility (nursing home), (2) unit, and (3) individual (care providers, care managers, and residents). Facility- and unit-level structural data were collected from facility administrators and care managers respectively using standardized profile forms developed for the TREC study. Individual resident-level data came from the Resident Assessment Instrument-Minimum Data Set Version 2.0 (RAI-MDS 2.0) administrative databases. We collected individual data from healthcare aides, nurses, physicians, allied health providers, practice specialists, and care managers, using the TREC survey which contains the ACT instrument as its first component. The TREC survey also contains components that measure: organizational context, knowledge translation (defined as uptake of research evidence or best practices), and staff outcomes (e.g., burnout, job satisfaction). We invited all individuals in the identified respondent groups who met the TREC study inclusion criteria (see Additional File 1) and who could be contacted to participate by completing the TREC survey. Research assistants administered the survey to healthcare aides (the dominant direct care provider group in Canadian nursing homes) using computer-assisted, structured personal interviews. The remaining staff groups completed the survey online. The core of the survey is the Alberta Context Tool (ACT); we used data from individual healthcare aides in the analyses reported here.

### Ethics

Ethical approvals for the TREC study were obtained from the appropriate universities in the respective Canadian Prairie Provinces (University of Alberta Health Research Ethics Board, University of Calgary Conjoint Health Research Ethics Board, University of Saskatchewan Behavioural Research Ethics Board, University of Manitoba Fort Garry Campus Research Ethics Board). Operational approvals were obtained from all relevant healthcare organizations.

### Data Analysis

#### Reliability

To assess the reliability of individual scores obtained from the healthcare aides, we calculated Cronbach's alpha for each concept contained in the ACT. Coefficients can range from 0 to 1; a coefficient of 0.70 is considered acceptable for newly developed scales, 0.80 or higher is preferred [17,18].

#### Acceptability

We assessed acceptability of the ACT with the healthcare aides in our sample by evaluating: (1) missing response rates for all ACT items combined, and (2) the average length of time it took to complete the ACT portion of the TREC survey.

#### Validity

Our approach to assessing validity builds on the perspective of construct validity outlined by Cronbach and Meehl [19], which has been incorporated into the Standards for Educational and Psychological Testing (the *Standards*) [20]. Its use is considered best practice in psychometrics [21]. Using this approach, validation is a process that involves accumulating evidence to provide a strong scientific basis for proposed score interpretations. Evidence for validity in the *Standards *comes from four sources: (1) content-the extent to which items represent the content domain of the concept of interest; (2) response processes-how respondents interpret, process, and elaborate on item content and whether this is in accordance with the concept; (3) internal structure-associations among items and whether the data supports the relevant dimensionality; and (4) relations to other variables-the nature and extent of the relationships between scores obtained for the concept and other variables to which it is/is not expected to relate. In previous research we established: (1) content validity of the ACT [10,22], (2) response processes evidence [10,22,23] and (3) early internal structure (principal components analysis) evidence in different sectors [10,22,23], including the nursing home sector [23]. In this paper we focused on: validity evidence type 3 - advanced aspects of internal structure, and validity evidence type 4 - relations with other variables, when completed by healthcare aides in nursing homes.

##### Internal Structure

We examined the internal structure of the ACT concepts using: (1) item-total statistics (using PASW Version 18.0 [24]) and (2) confirmatory factor analysis (CFA) (using LISREL [25]). From the item-total statistics, we considered items for further assessment if: (1) they correlated with the total scale (concept) score below 0.3, and (2) they caused a substantial rise or fall in Cronbach's alpha for the concept if removed [17,26]. We used a confirmatory approach to factor analysis to validate the latent structure of the ACT, which was refined in our previous work conducted in the pediatric setting [10]. The items included under each ACT conceptual dimension were designed to tap similar yet explicitly non-redundant contextual features, and hence the factor-structured models traditionally employed to assess internal structure are not precisely correct, though the similarity of items within the ACT conceptual dimensions renders the factor structure the most appropriate of the available model structures. We ran three factor models. Model 1 was comprised of all ACT items, the structure of which had been refined in our previous work in the pediatric setting. When Model 1 failed to function as anticipated, we did a more detailed investigation by setting up separate factor-structured models for the 7 scaled ACT concepts (Model 2) and the 3 non-scaled or count-based ACT concepts (Model 3).

Recent discussions on structural equation model testing [27,28] argue that the χ^2 ^statistic is the only reliable test of model fit, and question the use of commonly accepted fit indices such as the root mean square error of approximation (RMSEA), the standardized root mean squared residual (SRMSR), and the comparative fit index (CFI). While we tend to agree with the critiques of the fit indices, we are hesitant to entirely disregard them due to their previous common use (e.g., [29-31]). Consequently, we report the χ^2 ^test of model-data fit and the fit indices indicated above, though we are mindful that none of these are definitive for our current analyses given the intentional inclusion of non-redundant items within each ACT concept.

##### Relations to Other Variables

We assessed *relations to other variables *validity by providing the bivariate associations (Pearson's correlation coefficient) between the 10 ACT concepts and instrumental research utilization (which the ACT should predict). To permit correlating of the 10 ACT concepts with instrumental research use, we created a single score for each of the ACT dimensions by averaging the relevant items if the items were scaled (leadership, culture, evaluation, social capital, organizational slack-staff, organizational slack-time, organizational slack-space), or recoding the items as existing and non-existing and then summing the number existing if the items were part of a count-based measure (informal interactions, formal interactions, structural and electronic resources). As a second (related but more detailed) test of relations to other variables validity, we examined whether the mean values for each ACT concept increased with increasing levels of instrumental research utilization, and we assessed the mean differences for statistical significance using one-way analysis of variance (ANOVA).

Instrumental research utilization refers to a direct and concrete use of research evidence in practice (e.g., use of guidelines). In the TREC survey we defined instrumental research use as 'use of best practices' for the healthcare aides, and measured it with a single item scored on a 5-point frequency scale from 1 (never use) to 5 (use almost always). In a recent systematic review of the psychometric properties of self-report research utilization instruments, Squires et al [32] reported that this specific measure of instrumental research utilization has been used in eight published studies (reported in 10 articles) with professional nurses (n = 8 articles, [33-40]), healthcare aides (n = 1 article, [41]), and allied professionals (n = 1 article, [42]) across a variety of healthcare settings. Validity evidence from all three applicable sources of validity (content, response processes, and relations to other variables) outlined in the *Standards for Educational and Psychological Testing *[20] was reported in one or more of these 10 articles. In addition to this validity evidence from past studies, we also pre-tested the Instrumental Research Utilization item alongside the ACT before using it in the larger TREC study reported in this paper [23]. The sample for the pre-test included 73 healthcare aides and 18 licensed practical nurses from two nursing home units in one Canadian province.

##### Aggregation

The research team developed the ACT to permit unit and/or organizational level measurement of context, depending on the context of care delivery of the individuals completing the instrument. We hypothesized that in the case of healthcare aides in nursing homes the resident care unit constituted a relevant organizational feature. Therefore, as a final assessment of validity in this study, we calculated commonly used aggregation indices to assess the appropriateness of aggregating individual responses from healthcare aides on the ACT to higher (care unit and nursing home) levels. ANOVA was used to assess each of the 10 ACT derived concept scores using the care unit and the nursing home as grouping variables. The source table from this analysis was then used to calculate the following four aggregation indices.

1. **Interclass correlation ICC(1) **is a measure of agreement about the group mean. It is calculated as follows: (BMS - WMS)/(BMS + [K - 1] WMS), where BMS is the between-group mean square, WMS is the within-group mean square, and K is the number of subjects per group. The average K for unequal group size was calculated as K = (1/[N - 1]) (∑K - [∑K^2^/∑K]). Values greater than 0.00 indicate some degree of agreement among group members; values greater than 0.10 indicate strong agreement [43].

2. **Interclass correlation ICC(2) **is a measure of reliability. It is calculated as follows: (BMS - WMS)/BMS. Aggregated data are considered reliable when the ICC(2) is greater than 0.60 and/or the F value from ANOVA is significant [43].

3. **η^2 ^**is a measure of validity; it is an indicator of effect size and refers to the proportion of variation in the concept accounted for by group membership [44]. It is calculated as follows: SSB/SST, where SSB is the sum of squares between groups and SST is the sum of squares total.

4. **ω^2 ^**is a measure of validity; it measures the relative strength of the aggregated data (or score) at the group level [45] and indicates how much information is carried up from the individual level to the group level when the data (or scores) are aggregated. It is calculated as follows: (SSB - [N - 1] WMS)/(SST + WMS).

Larger values of η^2 ^and ω^2 ^indicate stronger validity of the aggregated data.

## Results

### Sample Characteristics

Within the TREC urban nursing home sample, a total of 1367 healthcare aides (representing 73% of those eligible to participate) completed the TREC survey in year one (July 2008-June 2009). For psychometric testing reasons, we desired a homogeneous sample and a sample from facilities with multiple units. Therefore, we conducted the analysis reported in this paper on a subsample of the 1367 healthcare aides as follows. We analyzed ACT scores from healthcare aides: (1) from 25 of the 30 urban nursing homes (i.e., all participating urban nursing homes that contained more than one resident care unit), and (2) where English was the first language of the healthcare aides. The final sample size consisted of 645 healthcare aides. Demographic characteristics of the healthcare aide sample are presented in Table 2.

**Table 2:** Characteristics of the Healthcare Aide Sample (n = 645)

Demographic Characteristic		n (%)
Gender	Male	32 (5.0)
	
	Female	609 (94.4)
	
	*Missing Values*	4 (0.6)

Age	< 20 years	10 (1.6)
	
	20-29 years	100 (15.5)
	
	30-39 years	111 (17.2)
	
	40-49 years	199 (30.9)
	
	50-59 years	162 (25.1)
	
	60-69 years	62 (9.6)
	
	> 70 years	0

	*Missing Values*	1 (0.2)
	
Shift worked most of the time	Day Shift	339 (52.6)
	
	Evening Shift	203 (31.5)
	
	Night Shift	103 (16.0)
	
	*Missing Values*	0

Education Level^1^	High school	563 (87.3)
	
	HCA Certificate	540 (83.7)
	
	Other Diploma/Degree	231 (35.8)



		**Mean (SD)**

Number of Years Worked as HCA		11.87 (9.78)

Number of Years Worked on Unit		4.86 (5.65)

Hours worked in 2 weeks		65.76 (17.95)

^1 ^= individuals may fall into more than 1 category, therefore the sum of values is greater than 100%

### Reliability

Table 3 displays the Cronbach's alpha coefficients for each of the 10 ACT concepts. Coefficients ranged from a low of 0.37 (for formal interactions) to a high of 0.92 (for organizational slack-staff). With the exception of two concepts (formal interactions, alpha = 0.37; and organizational slack-space, alpha = 0.64), reliability of all ACT concepts exceeded the accepted standard of 0.70 recommended by Nunnally and Bernstein [17] and Altman and Bland [46].

**Table 3:** Item Characteristics (n = 645)

Survey Concept	No. Items	No. Completed Responses	Mean Response	Standard Deviation	Reliability		
					
					Item-total Correlation (Range)	Cronbach Alpha	Item-total statistics (Alpha after an item deleted)
**Leadership**	6	638	3.87	0.68	0.565 - 0.700	0.86	0.82 - 0.85

**Culture**	6	639	3.83	0.56	0.370 - 0.516	0.72	0.66 - 0.71

**Evaluation**	6	638	3.44	0.64	0.213 - 0.641	0.74	0.66 - 0.78

**Social Capital**	6	638	4.01	0.51	0.357 - 0.520	0.72	0.66 - 0.71

**Informal Interactions**	9	635	4.06	1.56	0.260 - 0.554	0.73	0.68 - 0.74

**Formal Interactions**	4	641	1.32	0.73	0.092 - 0.308	0.37	0.23 - 0.43

**Resources**	11	637	2.68	1.72	0.126 - 0.469	0.70	0.65 - 0.72

***Organizational Slack***							
**Staff**	3	644	2.27	1.12	0.788 - 0.877	0.92	0.85 - 0.92
**Space**	3	642	3.14	1.03	0.134 - 0.680	0.64	0.21 - 0.87
**Time**	4	641	3.08	0.81	0.581 - 0.696	0.83	0.77 - 0.82

### Acceptability

We determined acceptability by assessments of: (1) missing values on the ACT items, and (2) time to complete the survey. The percentage of healthcare aides providing complete data on all 58 ACT items (i.e., with no missing data) was high at 93.5% (n = 603 of 645 healthcare aides). The mean time for completion of the ACT instrument section of the TREC survey in the sample reported in this paper was 11.08 minutes (standard deviation: 2.93 minutes), close to our goal of 10 minutes. Combined, these findings make the ACT an acceptable instrument for health services researchers wishing to obtain quantitative measurement of organizational context in nursing homes.

### Validity

#### Internal Structure

##### Item Total Correlations and Statistics

The ranges of corrected item-total correlations and item-total statistics, along with the means (and standard deviations), for each ACT concept, are displayed in Table 3. Most (51 of 58) corrected item-total correlations were greater than the predetermined cut-off of 0.3 indicating that in general, item scores within each concept were related to the overall score for that concept. The seven items that did not meet this minimal cut-off represented five ACT concepts: (1) evaluation (item *discuss data informally*, 0.213); (2) informal interactions (item *hallway talk*, 0.260); (3) formal interactions (item *change of shift report*, 0.092; item *team meetings*, 0.257); (4) structural and electronic resources (item *use of a computer hooked to the internet*, 0.264; item *attending in-services*, 0.126); and (5) organizational slack-space (item *adequate space for resident care*, 0.134). Item-total statistics (alpha after item deletion) for each concept remained relatively unchanged, with the exception of one concept: item *adequate space for resident care *(concept of organizational slack-space); if this item was deleted, alpha increased substantially from 0.64 to 0.87. Based on the item analysis summarized above, we retained all 58 ACT items for entry into the initial factor model (Model 1).

##### Confirmatory Factor Analysis

We tested three factor models. The χ^2 ^test statistic and fit indices for all three models are presented in Table 4. We started by testing a 10-factor model in which each of the 58 ACT items loaded onto 1 of the 10 corresponding ACT conceptual dimensions (Model 1). Though this model displayed fit indices that historically might have been described as close fit (see Table 4), the χ^2 ^test did not support fit (χ^2 ^= 4674, *df *1550, p < 0.00). Examination of the standardized residuals showed substantial misfit for 5 items of 2 of the 10 ACT concepts: (1) structural and electronic resources (4 items), and (2) organizational slack-space (1 item). The misfit was revealed in significant standardized residuals between these items and the remaining items comprising the ACT concept in question (structural and electronic resources and organizational slack-space, respectively). Hence we removed these items for the remaining analyses. The factor loadings for Model 1 (not shown) overall were moderate and in the direction hypothesized; however, loadings were stronger for the scaled concepts (i.e., where a mean of several items could be used to obtain a derived concept score) than in the remaining concepts, which were lists of items that were counted to obtain a derived concept score.

**Table 4:** Model-Data Fit

Model	Description	Degrees of Freedom	Chi-Square (P value)	RMSEA	SRMR	CFI
Model 1	All ACT Concepts	1550	4674 (0.000)	0.06	0.07	0.91

Model 2	ACT Scale Concepts	474	933 (0.000)	0.04	0.04	0.98

Model 3	ACT Non-Scale Concepts	167	828 (0.000)	0.09	0.08	0.85

This difference in item scaling led to testing two alternative models: Model 2 examined the ACT *scaled *concepts, and Model 3 examined the ACT *non-scaled *or *count-based *concepts. Model 2 contained 7 of the 10 ACT concepts (leadership, culture, evaluation, social capital, organizational slack-staff, organizational slack-space, and organizational slack-time). Based on the standardized residuals from Model 1 and the item-total correlations, we removed the item of availability of adequate space for residents, leaving 33 items in Model 2. Model 3 contained the three remaining ACT concepts (informal interactions, formal interactions, and structural and electronic resources). The four items of structural and electronic resources that revealed misfit based on the standardized residuals from Model 1 were removed, leaving 20 items in Model 3. We hypothesized a better fit would result for Model 2 compared to Model 3 because the items contained in Model 3 were developed to reflect a 'list' of items likely to have even less dependence on a common cause than the scaled items comprising Model 2. As expected, the intentional non-redundancy of items within all the ACT dimensions continued to be detected by χ^2^, but as predicted the χ^2 ^and fit indices were substantially better for Model 2 compared to Model 3 (see Table 4). Factor loadings in Model 2 were moderate to high and in the direction predicted. The loadings in Model 3 were also in the direction predicted, but were smaller in magnitude. The factor loadings for Models 2 and 3 are presented in Table 5.

**Table 5:** Completely Standardized Factor Loadings for ACT Concepts (Models 2 and 3)

Model	Concept	Item	Factor Loadings	
			Model 2	Model 3
**Model 2** **ACT Scaled Concepts**	**Leadership**	Looks for feedback Focuses on successes Calmly handles stress Listens, acknowledges, responds Actively mentors and coaches Resolves conflicts	0.652 0.622 0.744 0.771 0.761 0.691	
	
	**Culture**	Receive recognition Control over work Organization balances Professional development Clear on what patients want Supportive work group	0.558 0.588 0.621 0.585 0.566 0.445	
	
	**Evaluation**	Routinely receive information Discusses data informally Formal process Formulates action plans Monitors our performance Compares our performance	0.622 0.187 0.670 0.789 0.797 0.337	
	
	**Social Capital**	Share information with others Group participation is valued Information is shared Aim is to help others Observations are taken seriously Comfortable talking in authority	0.573 0.624 0.397 0.561 0.655 0.514	
	
	**Organizational Slack - Staff**	Get the *necessary w*ork done Deliver best possible care Have best day	0.824 0.946 0.907	
	
	**Organizational Slack - Space**	Private space Use of private space	0.874 0.904	
	
	**Organizational Slack - Time**	Do something extra for patients Look something up Talk about best practices Talk to someone about care plan	0.645 0.751 0.792 0.781	

**Model 3** **ACT Non-Scaled (Count-Based) Concepts**	**Informal Interactions**	Other healthcare aides LPNs RNs/RPNs Physicians Other healthcare providers Clinical educator/instructor Someone who brings new ideas 'Hallway talk' Informal bedside teaching		0.311 0.436 0.432 0.527 0.671 0.617 0.571 0.209 0.465
	
	**Formal Interactions**	Team meetings Resident rounds Family conferences Continuing education		0.606 0.552 0.110 0.297
	
	**Structural/Electronic Resources**	Library Textbooks Journals Notice boards Policies and procedures Clinical practice guidelines In-services		0.433 0.593 0.634 0.560 0.692 0.704 0.316

#### Relations to Other Variables

The correlations among the latent factors in Models 2 and 3 provide evidence that the variables corresponding to the various ACT concepts are functioning appropriately. The 10 ACT concepts are supposed to be distinct or non-redundant and hence they should not correlate overly highly with one another, though it is reasonable to presume that these dimensions might be somewhat coordinated due to real (but currently un-researched) causal forces operating in nursing home settings. Thus, appropriately functioning items should result in factor correlations that might vary substantially between the ACT concepts but that should not be extremely high. In Model 2 the latent (concept) level correlations ranged between 0.082 and 0.735, and in Model 3 from 0.398 to 0.615, providing evidence that the items appropriately differentiated between the intended conceptual dimensions.

As another way to assess 'relations to other variables validity', we examined associations (Pearson's correlation coefficients) between the 10 ACT concepts (using item means or item sums as appropriate, without the items that had been deleted from the factor models) and instrumental research utilization - a variable that we expected to depend on the contextual features measured by the ACT. Instrumental research utilization was significantly and positively correlated with 8 of the 10 ACT concepts (ranging from 0.111 for leadership to 0.199 for organizational slack-time). The two exceptions for which significant correlations were not noted were organizational slack-staff, and organization slack-space (see Table 6).

**Table 6:** Validity Assessment for Relations with Other Variables: Correlation of ACT Concepts with Instrumental Research Utilization (IRU) and Increasing Mean Values of the ACT Concepts by Increasing Levels of IRU

ACT Concept	Bivariate correlation **with IRU**^2^	**Mean value (and relative % change**^1^**) of ACT concepts by level of instrumental research utilization**						P-value for **mean diff**^3^
			
		1 n = 5	2 n = 11	3 n = 59	4 n = 263	5 n = 332	Total^†^	
Leadership	0.111**	4.33 (11.98)	3.70 (-4.47)	3.68 (-4.95)	3.80 (-1.79)	3.96 (2.26)	3.87	0.005

Culture	0.154**	3.90 (1.74)	3.58 (-6.72)	3.59 (-6.37)	3.80 (-0.86)	3.91 (1.99)	3.83	0.000

Evaluation	0.119**	3.23 (-6.11)	3.02 (-12.45)	3.30 (-4.23)	3.43 (-0.35)	3.50 (1.55)	3.44	0.030

Social Capital	0.174**	3.97 (-1.16)	3.79 (-5.61)	3.82 (-4.76)	3.96 (-1.34)	4.10 (2.13)	4.01	0.000

Informal Interactions	0.167**	3.10 (-23.57)	3.82 (-5.86)	3.40 (-16.09)	3.95 (-2.73)	4.28 (5.60)	4.06	0.001

Formal Interactions	0.124**	0.50 (-62.09)	0.91 (-31.08)	1.24 (-6.20)	1.28 (-2.61)	1.38 (4.98)	1.32	0.015

Structural/Electronic Resources^4^	0.141**	1.70 (-36.64)	2.27 (-15.29)	1.97 (-26.40)	2.67 (-0.58)	2.85 (6.40)	2.68	0.002

Organizational Slack - Staff	0.061	2.20 (-2.89)	2.00 (-11.72)	2.15 (-5.24)	2.22 (-1.87)	2.33 (2.86)	2.27	0.610

Organizational Slack - Space^5^	0.067	2.93 (-6.49)	2.91 (-7.27)	2.99 (-4.55)	3.11 (-0.81)	3.19 (1.83)	3.14	0.562

Organizational Slack - Time	0.199**	2.95 (-4.16)	2.86 (-6.96)	2.69 (-12.45)	2.97 (-3.46)	3.24 (5.23)	3.08	0.000

^†^Total = overall mean of the row concept

^1 ^= % of difference with respect to the total sample average

^2 ^= Pearson's correlation coefficients

^3 ^= P-value for one-way ANOVA using 5 IRU values

^4 ^= Score derived based on 7 items (excluding the 4 items representing electronic recourses that showed misfit in Model 1)

^5 ^= Score derived based on 2 items (excluding the 1 item on space for resident care that showed misfit in Model 1)

* = Significance at 0.05 level

** = Significance at 0.01 level.

Table 6 also presents the means of each ACT dimension for respondents reporting various levels of instrumental research use. Too few respondents reported low levels of instrumental research use for the corresponding means to be statistically stable but the 97.6% of the responses having stable (in columns labeled 3, 4, and 5 in Table 6) means displayed clear and systematic increases for all 10 ACT concepts. These trends are most easily seen if expressed as the relative percent difference in mean scores (from the sample average); one-way ANOVA's showed these differences were significant for the same 8 of 10 ACT concepts displaying significant correlations. This analysis shows a positive incremental coordination between ACT dimensions and one important likely consequence of superior ACT context scores; namely, increasing levels of instrumental research utilization.

#### Aggregation

The ACT is intended to permit unit and/or organizational level assessments of context via aggregation of individual-level responses to the items comprising the ACT. Table 7 provides information supporting aggregation of healthcare aides' responses to the care unit level. The ICC(1) values were all greater than 0.0 and were even greater than 0.10 for 6 of the 10 concepts, indicating a degree of perceptual agreement among the healthcare aides about the various ACT contextual features of the resident care units in which they work. The majority of ACT concepts (8 of 10) showed statistically significant F values (p < 0.01) and/or ICC(2) values greater than 0.60, which indicate reliable measurements of the ACT concepts when individual healthcare aides' responses were aggregated to the care-unit level. However, η^2 ^and ω^2 ^were low to moderate in size suggesting that the aggregated ACT scores should be thought of as reporting unit means rather than unit consensus. At the unit level there can be considerable disagreement between individuals despite unit-wide acknowledgement that any specific ACT context dimension may be generally superior or inferior for that unit.

**Table 7:** Unit-Level Aggregation^† ^of the ACT Concepts

ACT Concept	BMS	WMS	ICC1	ICC2	η2	ω2	F	PROB^1^
Leadership	0.823	0.407	0.124	0.505	0.245	0.123	2.020	0.000

Culture	0.610	0.265	0.152	0.565	0.269	0.152	2.298	0.000

Evaluation	0.611	0.381	0.077	0.376	0.204	0.077	1.603	0.001

Social Capital	0.359	0.243	0.062	0.322	0.191	0.061	1.474	0.006

Informal Interactions	2.607	2.419	0.011	0.072	0.148	0.011	1.077	0.308

Formal Interactions	0.603	0.526	0.020	0.127	0.154	0.020	1.145	0.188

Structural/Electronic Resources^2^	5.351	2.561	0.131	0.521	0.251	0.131	2.089	0.000

Organizational Slack - Staff	3.894	0.848	0.332	0.782	0.421	0.329	4.591	0.000

Organizational Slack - Space^3^	3.489	0.684	0.362	0.804	0.448	0.360	5.097	0.000

Organizational Slack - Time	1.479	0.535	0.197	0.639	0.306	0.195	2.767	0.000

^† ^= The definitions of the table's statistics are presented in the text

^1 ^= Probability of the corresponding F value

^2 ^= Score derived based on 7 items (excluding the 4 items representing electronic recourses that showed misfit in CFA Model 1)

^3 ^= Score derived based on 2 items (excluding the 1 item on space for resident care that showed misfit in CFA Model 1)

Our analyses also provided support for aggregating healthcare aides' responses on the ACT dimensions to the nursing home level (see Table 8). The aggregation into larger groups made group differences easier to detect statistically via the F test, but the variation between the larger groups (nursing homes) was less pronounced than the variation between the smaller groups (care units within nursing homes) (η2, Tables 7 and 8). Overall the support for nursing home level aggregation was weaker in comparison to that for resident care unit aggregation, although this may be an artifact of lower variation between nursing homes than between care units within nursing homes.

**Table 8:** Facility-Level Aggregation^† ^of the ACT Concepts

ACT Concept	BMS	WMS	ICC1	ICC2	η2	ω2	F	PROB^1^
Leadership	1.507	0.424	0.091	0.719	0.122	0.088	3.555	0.000

Culture	1.202	0.278	0.115	0.769	0.145	0.111	4.324	0.000

Evaluation	0.975	0.391	0.055	0.599	0.089	0.053	2.496	0.000

Social Capital	0.659	0.244	0.062	0.630	0.096	0.060	2.704	0.000

Informal Interactions	3.408	2.407	0.016	0.294	0.053	0.015	1.416	0.091

Formal Interactions	0.762	0.528	0.017	0.307	0.053	0.016	1.444	0.079

Structural/Electronic Resources^2^	12.312	2.580	0.128	0.790	0.158	0.124	4.772	0.000

Organizational Slack - Staff	10.833	0.894	0.303	0.917	0.320	0.293	12.117	0.000

Organizational Slack - Space^3^	9.687	0.734	0.323	0.924	0.339	0.313	13.193	0.000

Organizational Slack - Time	4.118	0.530	0.209	0.871	0.232	0.202	7.769	0.000

^† ^= The definitions of the table's statistics are presented in the text

^1 ^= Probability of the corresponding F value

^2 ^= Score derived based on 7 items (excluding the 4 items representing electronic recourses that showed misfit in CFA Model 1)

^3 ^= Score derived based on 2 items (excluding the 1 item on space for resident care that showed misfit in CFA Model 1)

## Discussion

This study represents the first reported assessment of the ACT in either residential long-term care settings or with data provided by healthcare aides. We assessed reliability, acceptability, and validity of the ACT when completed by healthcare aides in nursing homes. To frame our validity assessment, we used the Standards for Educational and Psychological Testing which builds on Cronbach and Meehl's [19] perspective on construct validity. We focused on evidence from two of the *Standards' *four sources of validity evidence: internal structure and relations to other variables. In addition, we assessed the performance of the ACT concepts with individual responses aggregated to the level of the resident care unit; we did this because we developed the ACT as a unit-focused measure.

### English as a First Language

In line with previous studies [47,48], a substantial number (48%) of the healthcare aides who participated in the TREC study did not speak English as their first language. This provides challenges from a psychometric perspective because a homogenous sample is preferred for psychometric assessments such as confirmatory factor analysis. There is evidence to suggest that healthcare aides differ on several psychological concepts; for example, conceptual research utilization [49], job satisfaction and burnout [50,51], and by ethnicity (of which first language spoken is a component). We, therefore, limited this initial assessment of the ACT with healthcare aides in nursing homes to individuals who spoke English as their first language. In future research we will conduct additional psychometric assessments with healthcare aides who do not speak English as their first language.

### Reliability and Acceptability

The internal consistency of the ACT, in terms of Cronbach's alpha coefficients, was for the most part consistent with usual practice for measures intended to be used at the *level of the group*, or in our case, the resident care unit [46,52]. Only two concepts had unacceptably low reliabilities: organizational slack-space and formal interactions. Both of these ACT concepts have few items (3 and 4 respectively). Within the *organizational slack-space *concept, 1 of the 3 items showed substantial misfit in the item-total statistics and CFA. When this item was removed from the scale, however, alpha increased substantially from 0.64 to 0.87. The low alpha found with the *formal interactions *concept can be explained by the fact that the items contained within this concept represent a 'list' of items. The items were purposefully selected to be non-redundant with each other and therefore, we expected lower reliability, as the item set were not developed as a 'true factor model'.

At just over 10 minutes to complete and with few missing data, the ACT met our criteria of acceptability. The low missing data values may also be attributed to our administration method (computer assisted structured personal interview). Pilot testing conducted prior to the study demonstrated that missing data would have been much higher if we had used traditional paper and pencil survey administration [23]. Currently, we are conducting a study to further compare the computer assisted structured personal interview to the paper/pencil administration of the survey in nursing homes.

### Validity

#### Internal Structure

We originally selected the items comprising the ACT to cluster within basic conceptual domains. We also intentionally designed the items to be non-redundant so that each item focused on a slightly different feature of the respondent's work environment. The clustering of items within conceptual domains renders the factor model appropriate for assessing the ACT but the purposefully non-redundant nature of items within conceptual domains guaranteed that the ACT would not function perfectly as a factor model. In fact, the factor models we estimated functioned unexpectedly helpfully. We employed three factor models: Model 1 with the entire set of items, and Models 2 and 3 with just the scale and non-scale (or count-based) items, respectively. Model 1 pointed to four electronic resource items as being inconsistent with the other resource items. *Electronic resources *and *structural resources *may reflect two separate concepts in the nursing home environment. Alternatively, the electronic resource items may have performed poorly as items due to the uniformly low availability of, and access to, electronic resources for healthcare aides in nursing homes in general, and in the sampled nursing homes in particular.

Model 1 also clearly reported that one *organizational slack-space *item (adequate space for resident care) did not function consistently with the other items of organizational slack-space (availability of private space to discuss care and knowledge, and use of private space to discuss care and knowledge). It correlated negatively with these other items, had a low item-total correlation, alpha increased if this item was deleted, and this item displayed substantial misfit in the standardized residuals in Model 1. This suggests that this particular item may not be appropriate for use with healthcare aides-possibly due to the nature of their daily tasks. In our first report on the ACT in which we used data from pediatric acute care facilities and registered nurses, this item performed much better [10]. As predicted, Model 2 for the scaled concepts (with the space item on 'adequate space for resident care' removed from the organizational slack-space concept) performed better than either Model 1 (all items) or Model 3 (count-based concepts with the 4 electronic resource items removed from the structural and electronic resources concept).

A model appropriately acknowledging the non-redundancy of the items would require use of single-item indicated latent concepts, but such a model does not provide the kind evidence required by the *Standards*. A better model would be to simultaneously assess both measurement and latent structures using structural equation modeling. We are, however, missing some elements that our theoretical framework stipulates would be required to undertake a full assessment in this manner. The PARiHS framework developers argue that optimal implementation of research is achieved when optimal levels of context, facilitation and evidence are present. A full assessment of construct validity would then include measures of evidence and facilitation, in addition to context. In this study, we are focusing on organizational context and its direct and indirect effects on research uptake and resident and staff outcomes and do not have the needed measures of facilitation or evidence to test the full PARiHS model. While an assessment of the influence of context on research uptake is the next planned analysis, the PARiHS framework *a priori *suggests that we will have low explained variance and fit problems with a structural equation model because we have only a partial set of the essential components of the framework. A confirmatory factor analysis was therefore our next best choice at this stage with which to assess the internal structure of the ACT.

#### Relations to Other Variables

To test relations to other variables, we conducted two correlational analyses. First, we examined the correlation coefficients between the 10 ACT latent concepts produced in the confirmatory factor analyses. Model 2 (scaled ACT concepts with the space item on 'adequate space for resident care' removed from the organizational slack-space concept) and Model 3 (count-based concepts with the 4 electronic resource items removed from the structural and electronic resources concept) were used in this assessment. The latent (concept-level) correlations between the ACT concepts were low to moderate in magnitude, providing evidence that the variables corresponding to the 10 concepts were functioning appropriately. That is, they are functioning as distinct (non-redundant) concepts.

As a second test of relations to other variables, we examined bivariate correlations between the 10 ACT concepts and instrumental research use (which the ACT was designed to predict). The five items (one organizational slack-space item and four electronic resource items) showing misfit in the confirmatory factor Model 1 and removed from Models 2 and 3 were also removed from this analysis. We found statistically significant relationships between 8 of 10 ACT concepts and instrumental research use. That is, higher levels of research utilization were associated with more positive contextual conditions. Further analyses also showed a trend for each of the 10 ACT concepts, of increasing mean values from low to high levels of instrumental research use, commencing at scale point 3. These findings are consistent with the PARiHS framework's assertions about the role of a positive context in promoting greater uptake of research findings and provide additional empirical support for the construct validity of the ACT.

#### Aggregation

Our aggregation statistics indicate that in nursing homes healthcare aide responses on the ACT can be reliably aggregated to obtain a unit-level assessment of organizational context. This is consistent with our previous report in the context of pediatric nurses' scores [10]. As with the registered nurses in our pediatric sample, healthcare aides perform most of their work on a single unit, are aligned with that unit, and therefore are able to assess and report on common practices and experiences of the unit - causing them to respond similarly on items within the ACT (i.e., items asking about their unit). Support for aggregating healthcare aide responses on the ACT to the nursing home level was, as expected, weaker than the care unit level. This is consistent with healthcare aides' work practices and experiences being aligned more with the unit than the larger facility. The statistics were also to be expected given that larger aggregates of people are expected to vary less than smaller aggregations, and much less than individuals' responses.

The ACT scores can be used individually or they can be aggregated to at least the care unit level. Healthcare aides constitute the majority of direct care providers in nursing homes and as such are the individuals who spend the most direct care time with residents. Thus, if our intent is to plan to develop and implement interventions that influence resident care, the healthcare aide perspective is the most germane. We are collecting assessments from other providers (e.g., registered nurses, licensed practical nurses and managers closely aligned with the resident care unit), but we are aware these will provide differing perspectives and work remains to describe these and to hypothesize their existence.

## Limitations

Validation of a newly developed instrument such as the ACT is a longitudinal and multi-step process requiring numerous positive findings across a variety of applications and settings. The report here represents only the second stage of our validation efforts; additional validation studies are needed to establish the reliability and validity of the ACT in other samples and settings. A stronger assessment of construct validity will be possible when future studies, implementing measures of evidence and facilitation, enable us to simultaneously assess the measurement and latent structure of the ACT using structural equation modeling; these are planned.

## Conclusion

We developed the ACT to have three characteristics: (1) a theoretical basis, namely the PARIHS framework, (2) parsimony, using the fewest number of items possible to reduce completion time, and (3) items that reflected modifiable features of context. The characteristic of parsimony has an impact on performance using traditional psychometric criteria. The validation process in this study demonstrated additional empirical support for construct validity of the ACT. This is the first assessment of the ACT in residential long-term care settings or with healthcare aides, and our findings support the ACT as an acceptable measure of context in this sector. The overall pattern of the data was consistent with the structure hypothesized in the development of the ACT. Our findings add to early evidence for its generalizability, but should still be interpreted with caution. These results support the ACT as an appropriate measure for assessing context in nursing homes at the individual healthcare provider (healthcare aide) level, as well as at the unit level by aggregating healthcare aide responses to the level of the care unit. Caution should be used in including the five items showing misfit (i.e., the space item in the organizational slack-space ACT concept and four electronic resource items in the structural and electronic resources ACT concept) with healthcare aides until further assessments are made.

Within the *Standards *approach, validity is not derived from any one source at a point in time; rather, it is accumulated over time and across studies. In this study, we offer internal structure validity evidence and relations to other variables validity evidence, adding to the existing validity evidence from content (the extent to which items represent the content domain) and response processes (how respondents interpret, process, and elaborate on item content and whether this is in accordance with the construct) reported previously [10]. Follow-up studies are in progress in which we are assessing the ACT with a wide array of healthcare workers-nurses, allied healthcare providers and professionals, physicians, and specialists (e.g., educators), and care managers in long-term care (nursing home) settings. Additional information on the ACT is available from the lead author of this paper.

## Competing interests

The authors declare that they have no competing interests.

## Authors' contributions

CAE, PGN, and GGC participated in conceptualizing the TREC program and in securing the grant that provided it's funding. CAE, PGN, JES, and GGC participated in conceptualizing TREC Project One and in data collection. CAE and JES led the statistical analytic design presented in the manuscript. JES and LAH assisted in performing and in interpreting the statistical analysis. All authors contributed to drafting the manuscript and approved the final version.

## Pre-publication history

The pre-publication history for this paper can be accessed here:

http://www.biomedcentral.com/1471-2288/11/107/prepub

## Supplementary Material

Additional file 1 **TREC Study Inclusion and Exclusion Criteria**. This file contains a description of the inclusion and exclusion criteria used in the TREC study that supplied the data used for the analysis reported in this paper. Click here for file
