# Supplementary material for: Advancing the argument for validity of the Alberta Context Tool with healthcare aides in residential long-term care
Source: BMC Med Res Methodol. 2011 Jul 18;11:107. doi: 10.1186/1471-2288-11-107 (PMC3156800; doi:10.1186/1471-2288-11-107)
Supplement: Additional file 1 — TREC Study Inclusion and Exclusion Criteria. This file contains a description of the inclusion and exclusion criteria used in the TREC study that supplied the data used for the analysis reported in this paper. [file 1471-2288-11-107-S1.DOC]

**Additional File 1: TREC Study Inclusion and Exclusion Criteria**

**Facility Inclusion and Exclusion Criteria**

| **Facility Inclusion Criteria** | 1. Registered by the provincial government 2. 90% of residents over 65 3. Conduct RAI-MDS 2.0 assessment since September 2007 4. Facility operation conducted in the English language 5. Rural sites greater than 100 km (but less than 200 km) radius of Regina or Saskatoon, and with populations of 10,000 people or less 6. Urban facilities must be within designated health regions (i.e., Alberta – Edmonton, Calgary, or East Central; Manitoba – Winnipeg; Saskatchewan – Regina-Qu’Appelle or Saskatoon) 7. Stable or minimal level of organizational flux |
| --- | --- |
| **Facility Exclusion Criteria** | 1. Facilities integrated with acute care 2. Facilities with a sub-acute service 3. Rural facilities within the Capital Health Region (Edmonton, AB), Calgary Health Region (Calgary, AB), and Winnipeg Regional Health Authority (Winnipeg, MB) that reside in places with populations of 10,000 people or less 4. Rural facilities less than 100 km or greater than 200 km of Regina or Saskatoon (SK) 5. Facilities with less than 35 long-term care beds 6. Dementia special needs facilities 7. Facilities undergoing (or expected to undergo) a degree of organizational flux within the proposed five-year lifespan of the TREC program |

Care Provider (Staff) Inclusion and Exclusion Criteria

| **Healthcare Aides** | **Inclusion Criteria:**   1. Identify a unit within a facility where they have worked for at least 3 months and are working now 2. Work a minimum of 6 shifts per month on this unit.   **Exclusion Criteria:**   1. Healthcare Aide Student |
| --- | --- |
| **Nurses**  [Registered Nurses (RNs) and Licensed Practical Nurses (LPNs)] | **Inclusion Criteria:**   1. **LPNS -** identify a unit within a facility where they have worked for at least 3 months and are now working; **RNs** – identify a unit within a facility or a facility where they have worked for at least 3 months and are now working      1. **LPNs -** work a minimum of 6 shifts per month on this unit; **RNs** - work a minimum of 6 shifts per month on this unit or in this facility   **Exclusion Criteria:**   1. Licensed Practical Nurse/Registered Nurse Student 2. Nursing instructors whose primary role is supervising students |
| **Allied Healthcare Providers** | **Inclusion Criteria:**   1. Identify a facility in which they provide at least one third (i.e., at least 6 days a month) of their long-term care services   **Exclusion Criteria:**   1. Allied Healthcare Student 2. Allied instructors whose primary role is supervising students |
| **Physicians** | **Inclusion Criteria:**   1. Physicians who see 10 or more residents in a facility 2. The Medical Director of the facility.   **Exclusion Criteria:**   1. Physicians not currently seeing residents 2. Residents or medical students 3. Academic staff |
| **Practice Specialists** | **Inclusion Criteria:**   1. Identify a facility in which they provide at least one third (i.e., at least 6 days a month) of their long-term care services   **Exclusion Criteria:**   1. Academic staff 2. Clinical instructors whose primary role is supervising students |
| **Care Managers** | **Inclusion Criteria:**   1. Identify one facility in which they work more than 50% of the time. 2. Facility administrators when there is no care manager who is responsible for resident care (e.g. only one unit in the facility)   **Exclusion Criteria:**   1. Managers **not** responsible for resident care (e.g., dietary managers, materials management managers) |
